# Long noncoding RNA LINC01510 promotes the growth of colorectal cancer cells by modulating MET expression

**DOI:** 10.1186/s12935-018-0503-5

**Published:** 2018-03-21

**Authors:** Chaoqun Cen, Jian Li, Jingjing Liu, Mingshi Yang, Tianyi Zhang, Yu Zuo, Changwei Lin, Xiaorong Li

**Affiliations:** 1 Department of Gastrointestinal Surgery, The Third Xiangya Hospital of Central South University, Tongzipo Road, Changsha, 410013 Hunan People’s Republic of China; 2 Department of Emergency Medicine and Intensive Care Unit, The Third Xiangya Hospital of Central South University, Tongzipo Road, Changsha, 410013 Hunan People’s Republic of China; 3 Department of Nuclear Medicine, The Third Xiangya Hospital of Central South University, Tongzipo Road, Changsha, 410013 Hunan People’s Republic of China

**Keywords:** LINC01510, Long non coding RNA, Cell cycle, Cell growth, MET, Colorectal cancer

## Abstract

**Background:**

Abnormal expression of long non-coding RNA (lncRNAs) often facilitates unrestricted growth of cancer cells. Long intergenic non-protein coding RNA 1510, an enhancer lncRNA (LINC01510), a lncRNA enhancer is upregulated in colorectal cancer (CRC), and its expression might relate to MET as revealed by lncRNA microarray data. However, the potential biological role of LINC01510 and its regulatory mechanism in CRC remain unclear. Therefore, we investigated the involvement of LINC01510 in the proliferation of CRC cells.

**Methods:**

Microarray analysis, In situ hybridization, colony formation assay, MTT assay, Western blotting, quantitative RT-PCR and flow cytometry were applied. The two-tailed Student’s *t* test and analysis of variance or general linear model of single factor variable was used for statistical analyse.

**Results:**

In the present study, we found that LINC01510 was significantly upregulated in CRC tissues and cell lines. The LINC01510 expression level were associated with the clinicopathological grade and stage. Meanwhile, gain- and loss-of-function assays demonstrated that LINC01510 overexpression increased CRC cell proliferation, and promoted cell cycle progression from the G1 phase to the S phase. Further study indicated that LINC01510 was positively correlated with the expression of MET, and its effects were most likely at the transcriptional level.

**Conclusions:**

Taken together, our findings suggested that upregulation of LINC01510 contributes to the proliferation of CRC cells, at least in part, through the regulation of MET protein. LINC01510 could be a candidate prognostic biomarker and a target for new therapies in CRC patients.

## Background

Colorectal cancer (CRC) is the third most commonly diagnosed cancer in the world, with over 1.2 million new cases each year [1]. It is associated with a high mortality rate due to its rapid progression and advanced tumor presentation at the time of diagnosis [2, 3]. Despite of the therapeutic advances that have been made in CRC in the past few decades, the survival rate of CRC patients still remains unsatisfied [4]. The early detection of CRC is obviously beneficial to improve the probability of survival. Therefore, elucidating the mechanisms result in CRC and searching for some specific biomarkers associated with CRC that can be used to create novel diagnostic and therapeutic strategies is urgently needed.

Long non-coding RNAs (lncRNAs), sized from 200 nt to 100 kb, are a class of newly discovered non-coding RNA with limited or no protein-coding capacity [5, 6]. Accumulating evidence suggests that lncRNAs are key regulatory molecules that are involved in multiple biological processes, including chromosome inactivation, cell differentiation, genomic imprinting and development, and cell proliferation [7–9]. Recently, increasing evidence has shown that lncRNAs could be considered as a promising diagnostic tool and a convenient prognostic biomarker [10], for some of them are highly expressed in several types of cancer and their expression was associated with pathophysiological characteristics of tumor growth. For example, elevated expression of long noncoding RNA SPRY4-IT1 was reported in melanoma cells and its downregulation inhibited invasion and proliferation of melanoma cells [11]. Knock down of SPRY4-IT1 reduced renal cell proliferation, migration, and invasion [12]. A small number of lncRNAs have been identified for their association with the occurrence of cancer development [13–15], but more are waited to be identified and await functional validation in CRC.

We identified a new enhancer lncRNA, LINC01510 (ENST00000450063) was upregulated in CRC tissues and related to the expression of MET detected by microarray analysis. And MET has been found to be a tumor promoter in several types of cancer related to tumor growth, including CRC [16, 17]. However, the relation of LINC01510 and MET in CRC was unclear. So, in this study, we investigated the function of LINC01510 in CRC cells. The results of our study indicated that LINC01510 could influence cell proliferation and cell cycle distribution through regulating MET expression.

## Methods

### Microarray analysis

Briefly, samples (three pairs of CRC and para-carcinoma tissues) were used for LncRNA expression microarray, the microarray analysis was performed by Gminix, Shanghai, China.

### RNA extraction and qRT-PCR analyses

Total RNA was extracted from tissues or cultured cells with the TRIZOL reagent (Invitrogen Life Technologies, Carlsbad, CA, USA). For qRT-PCR, RNA reverse transcribed to cDNA from 1 μg of total RNA was reverse transcribed in a final volume of 20 μL using Oligo dT and a Reverse Transcription Kit (Fermentas, Thermo Scientific, Waltham, MA, USA). qRT-PCR analyses were performed using a standard protocol from SYBR Green qRCR Mix (TOYOBO, Osaka Prefecture, Osaka, Japan) on the ABI 7300 sequence system (Applied Biosystems, CA, USA). PCR was performed with the following thermocycling conditions: an initial of 3 min at 95 °C, followed by 40 cycles of 95 °C for 10 s, 60 °C for 30 s. All protocols were performed according to the manufacturer’s instructions. The relative expression level was calculated using the Ct method. Human β-actin was used as an internal control. Each sample was analyzed in triplicate. The primers used for each reactions were as follows: LINC01510 forward 5′-CTGTGGAAGTTTGAGTGAC-3′ and reverse 5′-TTCATCTATCCTCCTGCT-3′; MET forward 5′-CCGCTGACTTCTCCACTG-3′ and reverse 5′-TTCATCTCGGACTTTGCT-3′; SATB2-AS1 forward 5′-AGAAGCAAAGAATCATACTCCA-3′ and reverse 5′-ATGCCTAACTAAGAACGACAAA-3′; SATB2 forward 5′-AGGAGTTTGGGAGATGGTAT-3′ and reverse 5′-ACTGAACCTGACCGTACACCCAGAACACAATAGTCTGAA-3′; NR-026995 forward 5′-CTCCCAAGTAACCCTCTAA-3′ and reverse 5′-CACTCCCTGTCCTCAAAT-3′; RAPGEF4 forward 5′-5′TTTTATGCCAAATACCCAG-3′ and reverse 5′-CGGATGACTCGCCTCTTA-3′; β-actin forward 5′-AGGGGCCGGACTCGTCATACT-3′ and reverse 5′-GGCGGCACCACCATGTACCCT-3′.

### In situ hybridization (ISH)

CRC and normal tissues were fixed with 4% paraformaldehyde, dehydrated, embedded in paraffin, sectioned at 4 μm and mounted onto charged slides. After deparaffinization and hydration, slides were air dried and treated with 3% hydrogen peroxide in distilled water before applying pepsin solution for 30 min at 37 °C. Prehybridization was performed at 37 °C for 2 h in hybridization buffer. Slides were hybridized with target DIG-labeled probes (TCCAGATGAACTCCTCCTAC) overnight at 42 °C, followed by a series of washing steps (2 × SSC, 0.5 × SSC, 0.2 × SSC). Blocking buffer was applied for 30 min at 37 °C followed by biotinylated anti-mouse digoxin (Boster Biological Technology, Wuhan, China) for 1 h at 37 °C and then SABC-POD (Boster Biological Technology, Wuhan, China) for 20 min at 37 °C. Hybridization signals were detected by chromogenic reactions using DAB followed by hematoxylin solution counterstaining. After dehydration, slides were mounted neutral gum and examined by microscopy.

### Cell lines and tissues

Human colon epithelial cell NCM460, CRC cell lines HT-29, HCT116, SW620 and LoVo were purchase from cell bank of chinese academy of sciences (Shanghai, China). NCM460 was cultured in McCoy’s 5a supplemented with 10% fetal bovine serum (Gibco, California, USA). HT-29, HCT116, SW620 and LOVO were cultured in Dulbecco’s Modified Eagle Medium (Hyclone, Logan, UT, USA) supplemented with 10% fetal bovine serum. All cells were cultured at 37 °C in an atmosphere of 5% CO_2_. The CRC tissues and CRC and para-carcinoma tissues was collected from department of gastrointestinal surgery, The Third Xiangya Hospital of Central South University between April and October 2015. The tissues were obtained from patients with CRC via surgery. The study was approved by the institutional research ethics committee of the Third Xiangya Hospital of Central South University, and written informed consent was obtained from each subject.

### Plasmids construction and cell transfection

The LINC01510 or MET sequences were amplified from genomic DNA and cloned into the pcDNA3.1(+) vector. Base-pair oligos for hairpin RNA expression targeting LINC01510 was inserted into pRNAT-U6.1/Neo vector. The recombinant plasmids were identified by restrictive endonuclease enzymes digestion and sequencing. SW620 or LoVo cells were plated in 6-well plates at a density of 1 × 10^5^ cells/well and incubated at 37 °C until the cells were about 80% confluent before transfection. The recombinant plasmids were transfected into cells cultured in six-well plates using lipofectamine 2000 (Invitrogen, Carlsbad, CA, USA), according to the manufacturer’s instructions. The cells were harvested at the indicated time according to experimental requirements.

### MTT (3-[4,5-dimethylthiazol-2-ul]-2,5-diphenyltetrazolium bromide) assay

Cells transfected with recombinant plasmids according the experimental requirements were plated at 96-well plates at a density of 5 × 10^3^ cells/well. The MTT reagent (Sangon, Shanghai, China) added to each well at the indicated time points and incubated for 4 h at 37 °C. The optical density (OD) was read at 570 nm on a microplate spectrophotometer. Quintuplicate wells were measured in each treatment group.

### Colony formation assay

Cells were seeded in 6-well plates at a density of 400 or 200 cells/well. After incubation for 14 days, cells were washed twice with PBS, fixed with methanol and stained with crystal violet. The number of colonies containing > 50 cells was counted under a microscope.

### Western blotting

The cells in each group were lysed in RIPA lysis buffer (Auragene, Changsha, China) supplemented with a protease inhibitor cocktail and PMSF. The protein concentration was measured using BCA kit (Auragene). Proteins were separated by 80% sodium dodecyl sulfate–polyacrylamide gel electrophoresis (SDS-PAGE) and transferred onto polyvinylidene fluoride membranes (Invitrogen, Carlsbad, CA, USA). The membranes were blocked in 5% nonfat dry milk solution in TBS buffer plus 0.1% Tween 20 for 30 min at 37 °C and then incubated with diluted primary antibody overnight at 4 °C. Following three washes, membranes were then incubated with secondary antibody for 40 min at 37 °C in TBST. Anti-rabbit or anti-mouse immunoglobulin G antibodies conjugated to horseradish peroxidase (Auragene) were used as the secondary antibodies. An ECL chemiluminescence detection kit (Auragene) was used for detection of antibody-antigen complexes. A β-actin antibody was used as a control. Bax polyclonal antibody and CDK2 polyclonal antibody were purchased from immunoway biotechnology (Newark, DE, USA) and the anti-Bcl-2, caspase-3, P21, MET antibodies were purchased from Abcam (Abcam, Cambridge, UK). This assay was repeated three times, and the results was analyzed by IPP6.0 for three times.

### Flow cytometry

Cells transiently transfected with the indicated plasmids were harvested 48 h after transfection by trypsinization, washed with ice-cold phosphate-buffered saline, and fixed with 70% ethanol overnight. The cells were then collected by centrifugation and resuspended in PI solution (50 mg/mL in PBS) containing 0.25 mg/mL of RNase A. After incubation for 15 min in the dark at 4 °C, the cells were analyzed by flow cytometry (FACS Canto II, BD Biosciences, New Jersey, USA) using an instrument equipped with Cell Quest software (BD Biosciences). The percentages of the cells in G0–G1, S, and G2–M phases were counted and compared. This assay was repeated three times, and the results was analyzed for three times.

### Statistics

The values given are mean ± SD. P values were determined for experimental versus control treatments by two-tailed student’s t test and analysis of variance or general linear model of single factor variable. The two-tailed student’s t test was used for analysing significance of difference in two group. And analysis of variance was used for analysing significance of difference among three or more than three groups.*P < 0.05, **P < 0.01, ***P < 0.001 were considered to indicate a statistically significant difference.

## Results

### Expression of LINC01510 in colorectal cancer patients and cell lines

**Fig. 1:**
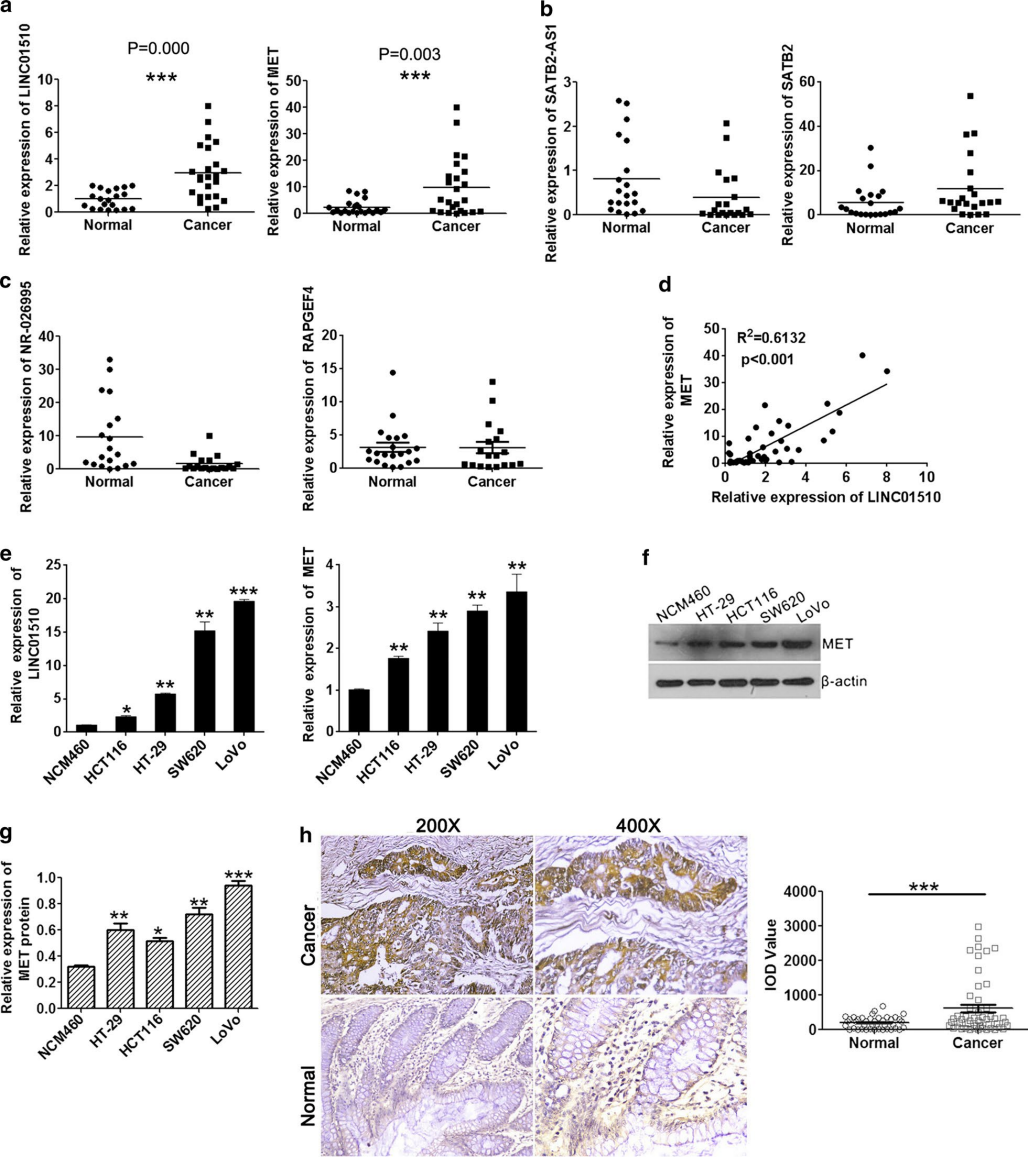
Relative expression of LINC01510 and MET in colorectal cancer tissues and cells. The relative RNA levels of LINC01510 and MET (**a**), SATBS-AS1 and SATB2 (**b**), NR-026995 and RAPGEF4 (**c**) in 20 pair CRC and normal tissues. **d** Correlation between expression level of LINC01510 and that of MET in 20 matched tissues. Linear regression coefficient and statistical significance is indicated. **e** The relative RNA level of LINC01510 and MET in different CRC cell lines and normal colorectal cells. **f**, **g** The protein level of MET in different CRC cell lines and normal colorectal cells. **h** RNA in situ hybridization assay was used to determination the expression of LINC01510. Data were based on at least three independent experiments and shown as mean ± SD. *P < 0.05, **P < 0.01, ***P < 0.001

The lncRNA microarray data showed that 79 lncRNAs were differentially expressed in CRC tissues compared with that in para-carcinoma tissue, consisting of 28 antisense lncRNAs, 51 enhancer lncRNAs and their corresponding mRNAs (< 300 Kb). All were analyzed and the top five-ENST00000415054, ENST00000517697, ENST00000230113, NR-026995 and LINC01510-were differentially expressed lncRNAs and were chosen for qRT-PCR assay in CRC and normal tissues and cells (Table 1). The results showed that ENST00000415054, ENST00000517697 and ENST00000230113 were not detected in cancer tissues because of their low abundance (data not shown). LINC01510 and MET level were upregulated in CRC tissues compared with those in normal tissues (Fig. 1a), which was consistent with microarray analysis. whereas SATB2-AS1 (Fig. 1b) and NR-026995 (Fig. 1c) levels were downregulated in CRC tissues compared with those in normal tissues, contrary to the results of microarray data. Moreover, LINC01510 was positively correlated with the expression of MET (Fig. 1d). therefore, we chose LINC01510 for further studies. We next examined the expression of LINC01510 and MET in four CRC cell lines (HCT116, HT29, SW620, LoVo) and a normal colonic epithelial cell line (NCM460) using qRT-PCR and Western blotting. As shown in Fig. 1e–g, the expression levels of LINC01510 and MET were indeed upregulated in all of the CRC cell lines examined compared with the normal cell lines. We chose to use LoVo and SW620 cell lines for the following experiments because they had the highest expression levels. Furthermore, in situ hybridization assay was performed to explore the result and identify the localization of LINC01510 in CRC and normal tissues. We found significantly higher expression of LINC01510 in CRC tissues than that in normal tissues and LINC01510 was expressed in cytoplasm (Fig. 1h). In addition, we analyzed the correlation between LINC01510 expression and the clinicopathological parameters present in present in patients with CRC. As shown in Table 2, the high-LINC01510 group showed a correlation with high grade and stage. However, there were no significant correlations between LINC01510 expression level and age. Taken together, these data suggest that LINC01510, an enhancer lncRNA, was upregulated in CRC related to high grade and stage and might upregulate the expression of MET.

**Table 1:** The most different expression of lncRNAs and their corresponding mRNAs analysed from lncRNA microarray data

LncRNA seqname	Gene symbol	Regulation	P value	FDR	Fold change	Gene	Associated_gene_acc	Regulation	Fold change
NR_026830	SATB2-AS1	Down	0.004	0.038	12.196	SATB2	NM_015265	Down	8.670
ENST00000415054	RP1-85F18.6	Down	0.004	0.038	13.351	EP300	NM_001429	Down	9.270
ENST00000517697	RP11-317J10.2 (CA3-AS1)	Down	0.003	0.036	5.581	CA2	NM_000067	Down	4.838
ENST00000230113	RP4-763G1.2	Down	0.003	0.036	9.176	GADD45A	NM_001924	Down	3.384
ENST00000450063	AC006159.3 (LINC01510)	Up	0.003	0.036	5.094	MET	NM_000245	Up	3.660
NR_026995	RAPGEF4-AS1	Down	0.002327827	0.046	81.1524549	RAPGEF4	NM_007023	Up	2.456277

**Table 2:** Correlationship between LINC01510 expression level and clinicopathologic characteristics in colorectal cancer tissues

Clinicopathological features	No cases	LINC01510 expression		*P* value
		Low	High	
Age (years)				
> 45	42	18	24	0.763
≤ 45	50	23	27	
Grade				
I	28	18	10	
II	30	12	18	0.035
III + IV	34	11	23	
T				0.011
1	24	17	7	
2	18	6	12	
3	50	18	32	

### Knockdown of LINC01510 inhibits cell proliferation in colorectal cancer

**Fig. 2:**
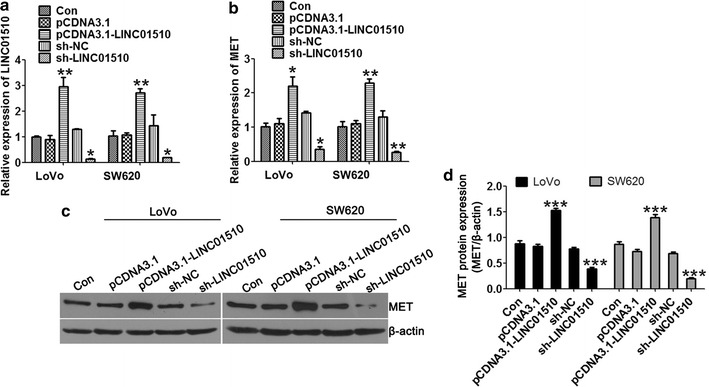
The effects of overexpression and knockdown plasmid of LINC01510. **a** Relative expression levels LINC01510 in LoVo and SW620 cells with LINC01510 overexpression and knockdown. **b** Relative expression levels of MET in LoVo and SW620 cells with LINC01510 overexpression and knockdown. **c**, **d** The protein expression of MET in LoVo and SW620 cells with LINC01510 overexpression and knockdown. Results are expressed as blot diagram (**c**) and gray intensity calculated expression of MET (**d**). Data were based on at least three independent experiments and shown as mean ± SD. *P < 0.05, **P < 0.01, ***P < 0.001

To investigate the effect of LINC01510 on CRC cell proliferation, first, pCDNA3.1-LINC01510 for LINC01510 overexpression and sh-LINC01510 for LINC01510 silencing were constructed and transfected in LoVo and SW620 cells, respectively. The transfection efficiencies were subsequently detected using qRT-PCR assays as shown in Fig. 2a, b. The mRNA expression level LINC01510 was effectively reduced after sh-LINC01510 transfection and elevated by pCDNA3.1-LINC01510 transfection compared with that in the control group in both cells. In addition, we detected the mRNA and protein expression levels of MET under the conditions of LINC01510 overexpression and silencing in LoVo and SW620 cells using qRT-PCR (Fig. 2c) and Western blotting (Fig. 2d). The mRNA and protein expression levels of MET were dramatically higher in the LINC01510 overexpression group than in the control group. In contrast, the mRNA and protein expression levels of MET were significantly decreased after knocking down LINC01510 expression. These results strongly indicated the association between LINC01510 and MET. MET was upregulated by LINC01510 at the transcription level.

**Fig. 3:**
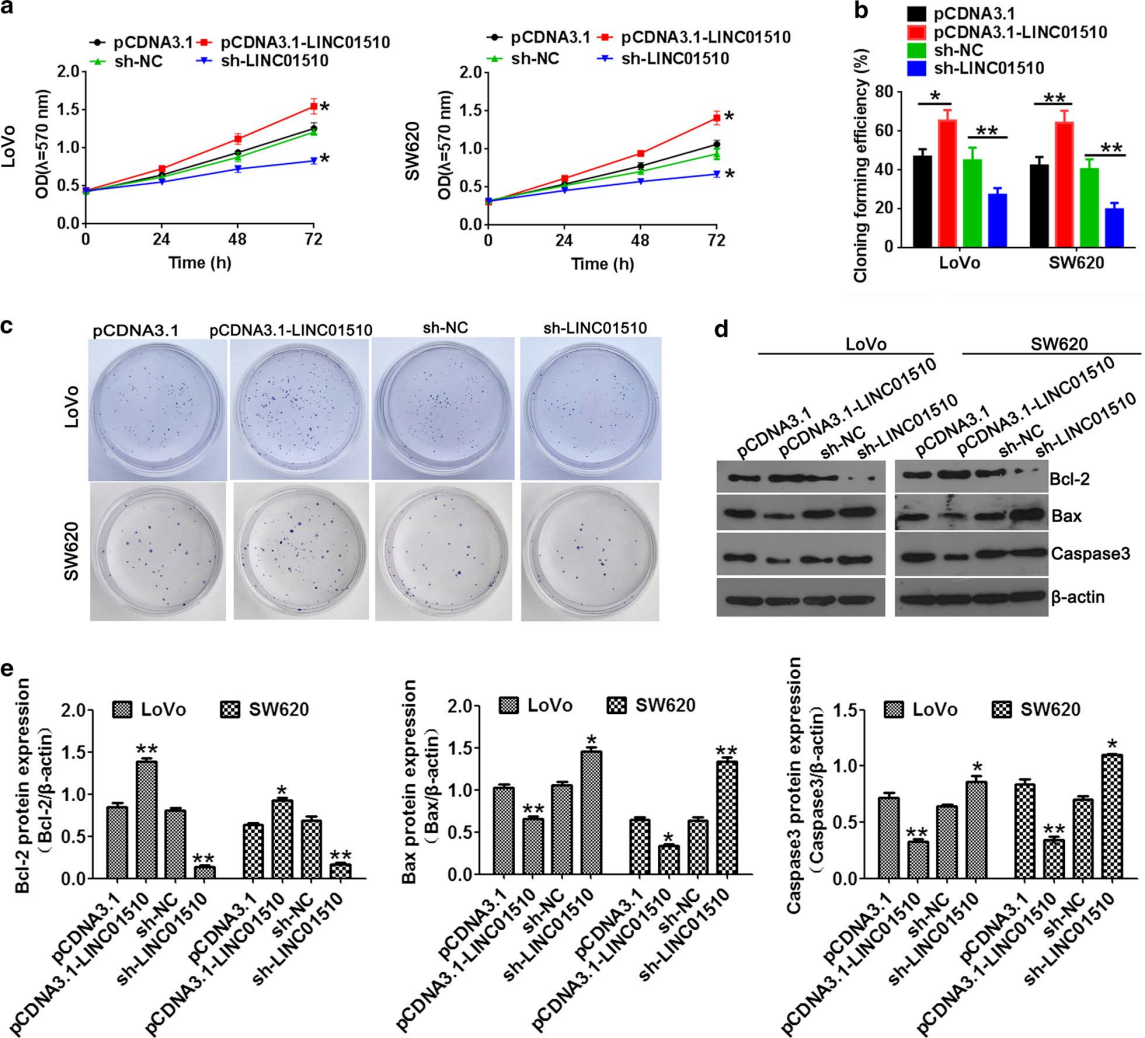
The effects of LINC01510 on cell proliferation in colorectal cancer cells. The cell growth was determined by MTT assay when LINC01510 overexpression and knockdown in LoVo and SW620 cells. **b** Statistical results of colony formation efficiency. **c** Colony formation assays were performed on LINC01510 up and down-regulation in LoVo and SW620 cells. **d**, **e** The apoptosis protein expression of Bcl-2, Bax and caspase3 in LoVo and SW620 cells with LINC01510 overexpression and knockdown. Results are expressed as blot diagram (**d**) and gray intensity calculated expression of Bcl-2, Bax and caspase3 (**e**). Data were based on at least three independent experiments and shown as mean ± SD. *P < 0.05, **P < 0.01, ***P < 0.001

Next, the biological role of LINC01510 on the proliferation in CRC cells was detected by MTT assay and clone formation assay. MTT assay revealed that LINC01510 overexpression obviously promoted cell proliferation of LoVo and SW620 cells (Fig. 3a). LINC01510 knockdown significantly inhibited cell proliferation. Similarly, the number of colonies obtained from LINC01510 overexpression cells was significantly higher than in the controls cells and dramatically lower in the LINC01510 downregulated cells than in the control cells (Fig. 3b, c).

To explore the potential mechanism of LINC01510-regulated cell growth, we detected the expression level of Bcl-2, Bax and caspase3 by Western blotting. Bcl-2, Bax, caspase3 are markers in the mitochondrial apoptotic pathway and play an important role in promoting cellular apoptosis [18–20]. As shown in Fig. 3d, e, the expression of Bcl-2 significantly increased in the LINC01510 overexpression group and declined in the LINC01510 knockdown group in both LoVo and SW620 cells. In contrast, the expression of Bax and caspase3 were dramatically decreased in the LINC01510 overexpression group and increased in the LINC01510 downregulated group in both cells. These results demonstrated that knockdown of LINC01510 exerted tumor-suppressive effects on human CRC cells.

### Knockdown of LINC01510 induced G0/G1-phase arrest in colorectal cancer cells

**Fig. 4:**
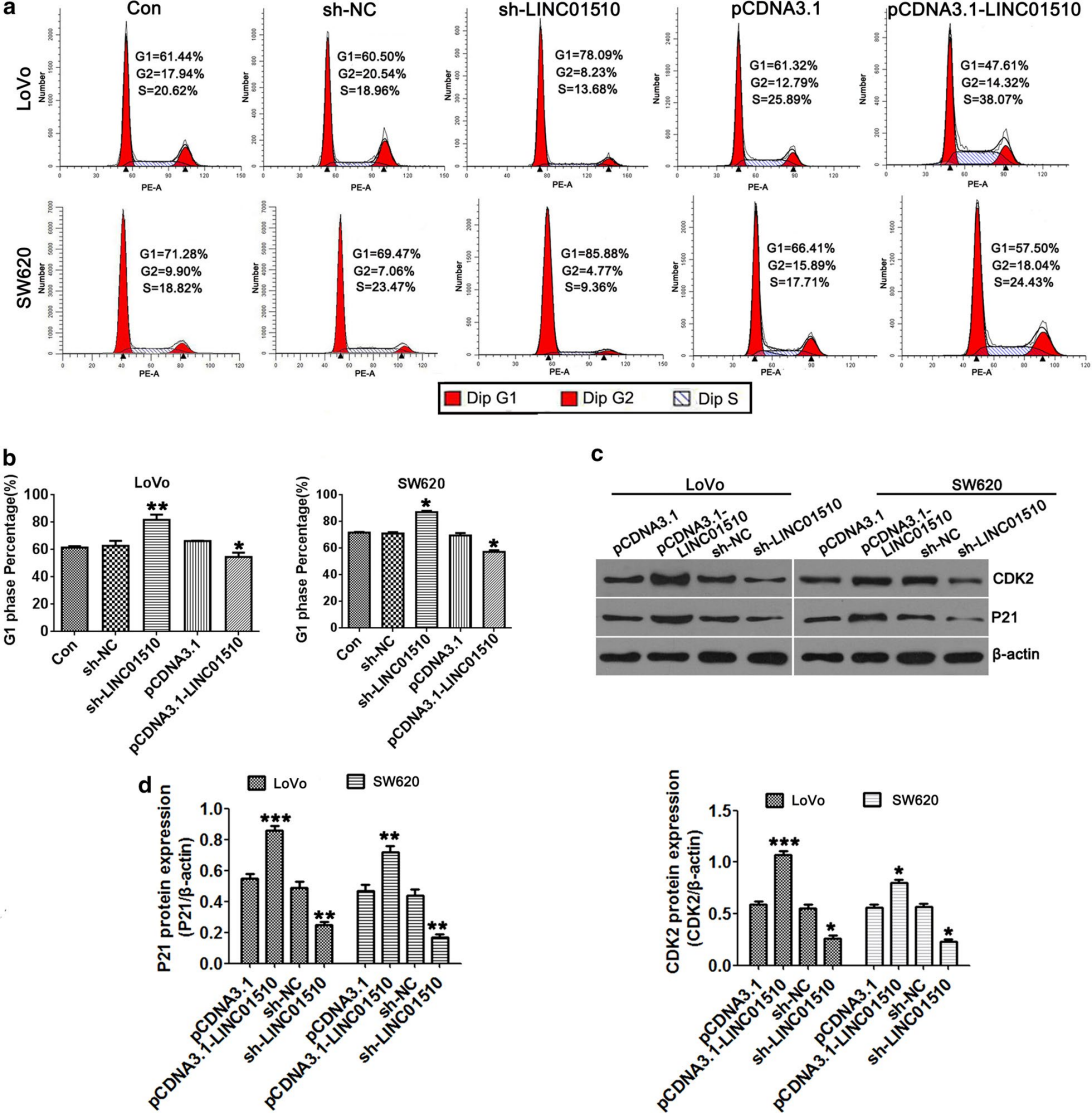
Knockdown of LINC01510 induced G0/G1-phase arrest in colorectal cancer cells. **a**, **b** Detection for cell cycle of CRC cells after silencing and overexpression of LINC01510 expression in LoVo and SW620 cells. Results are expressed as peak diagram (**a**) and calculated distribution for cells in G0/G1 phases (**b**). **c**, **d** The apoptosis protein expression of CDK2 and P21 in LoVo and SW620 cells with LINC01510 overexpression and knockdown. Results are expressed as blot diagram (**c**) and gray intensity calculated (**d**) expression of CDK2 and P21. Data were based on at least three independent experiments and shown as mean ± SD. *P < 0.05, **P < 0.01, ***P < 0.001

Cell cycle arrest reduces cell proliferation. We next analyzed the effect of LINC01510 knockdown and overexpression on cell cycle progression by flow cytometry and Western blotting. Compared with the control, Sh-LINC01510 led to a significant accumulation of cells in the G1-phase and an obvious decrease in the S-phase cells. LINC01510 overexpression led to a dramatic decrease of cells in the G1 phase along with an increase in the S-phase. These suggested that silencing of LINC01510 prevented G1 phase cells from entering the S phase (Fig. 4a, b).

To explore the molecular mechanisms by which LINC01510 inhibited cell cycle arrest (induced G0/G1-phase arrest) in CRC cells, the expression of S phase specific cell cycle regulatory proteins was investigated. P21, a downstream target of the P53 apoptosis pathway, inhibits cell proliferation by suppressing kinases that are critical for cell cycle progression [21, 22]. CDK2 binds to cyclin A to promote DNA duplication and the suppression of the expression of CDK2 often induced cell cycle arrest [23, 24]. As shown in Fig. 4c, d, LINC01510 overexpression treatment in LoVo and SW620 cells significantly increased CDK2 and P21 protein expression and suppressed CDK2 and P21 protein expression in the LINC01510-silenced treatment group. Taken together, knockdown of LINC01510 induced G0/G1-phase arrest in CRC cells.

### LINC01510 promotes proliferation of colorectal cancer cells through regulating the expression of MET

**Fig. 5:**
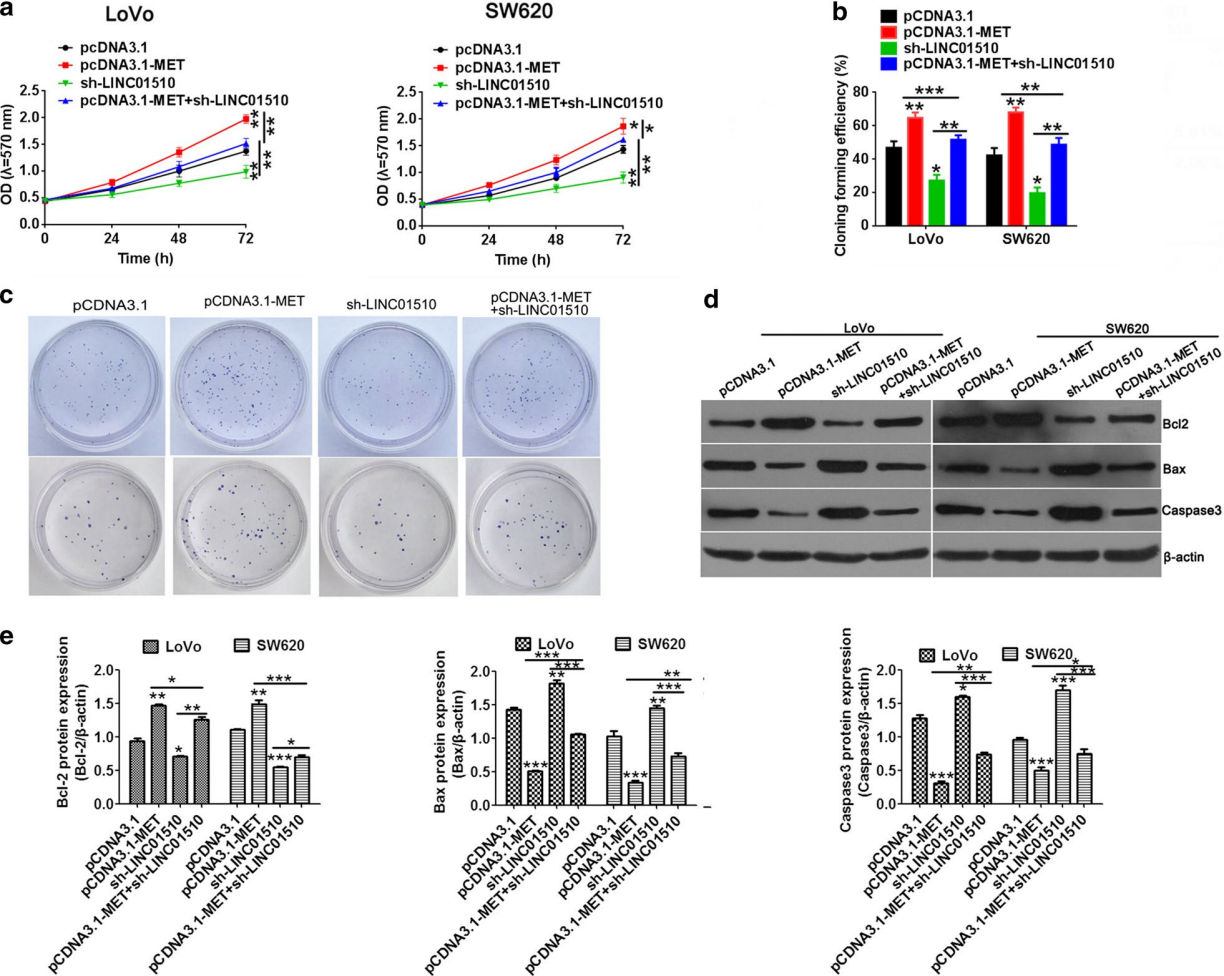
LINC01510 promotes cell growth of colorectal cancer cells through MET. **a** The proliferation of LINC01510 knockdown in LoVo and SW620 cells under MET overexpression, determined by MTT assay. **b** Statistical results of colony formation efficiency. **c** Colony formation assays were performed on LINC01510 down-regulation under MET overexpression in LoVo and SW620 cells. **d**, **e** The protein expression of CDK2 and P21 in MET overexpressing CRC cells under LINC01510 knockdown, measured by Western Blot assay. Results are expressed as blot diagram (**d**) and gray intensity calculated expression of Bcl-2, Bax and caspase3 (**e**). Data were based on at least three independent experiments and shown as mean ± SD. **P < 0.01, ***P < 0.001

To explore the exact mechanism by which LINC01510 promoting CRC cell proliferation, the relationship between LINC01510 and MET was examined. As shown above, LINC01510 could regulate the expression of MET at the transcription level, therefore, we hypothesized that LINC01510 might exert its function through the regulation of MET. To test this hypothesis, we performed MTT, clone formation assay and Western blotting assays. MTT and clone formation assays revealed that MET could significantly promote cell proliferation. MET overexpression compromised the effects of LINC01510 knockdown on cell inhibition (Fig. 5a–c) and apoptosis associated protein induction (Fig. 5d, e). These data indicated that LINC01510 promotes cell proliferation in a MET-dependent manner.

### LINC01510 promotes cell cycle arrest in G1 phase of in colorectal cancer cells by regulating the expression of MET

**Fig. 6:**
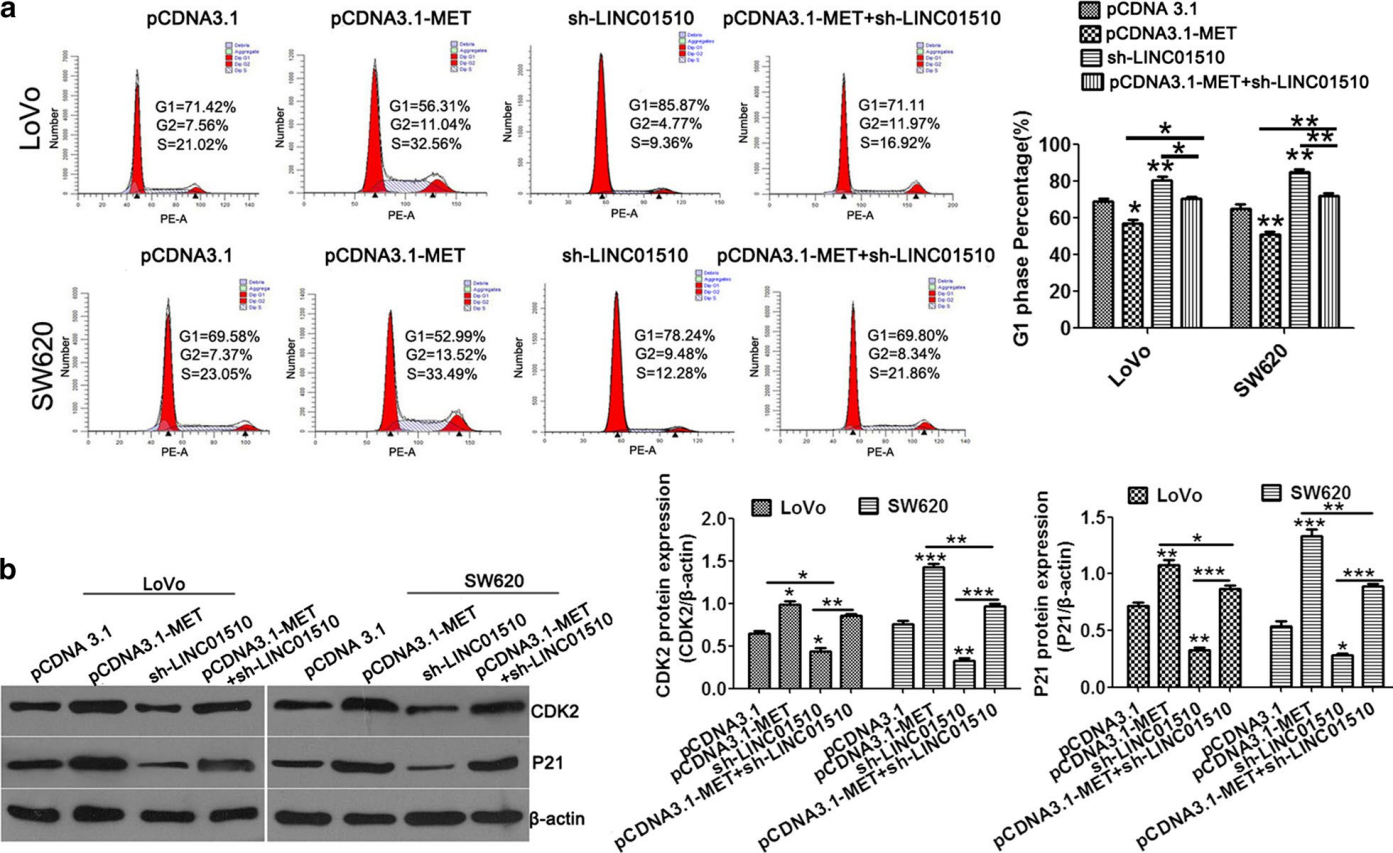
LINC01510 knockdown inhibits G1-phase arrested of colorectal cancer cells through MET. **a** LINC01510 silencing rescued the acceleration of the number of G1-Phase cells accumulation mediated by MET overexpression in LoVo and SW620 cells detected by flow cytometry. **b** The protein expression of Bcl-2, Bax and caspase3 in LoVo and SW620 cells with MET overexpression under LINC01510 knockdown, detected by Western blot assay. Data were based on at least three independent experiments and shown as mean ± SD. **P < 0.01, ***P < 0.001

Finally, we determined the function of LINC01510-MET association on cell cycle progression. Figure 6a shows that MET overexpression significantly led to cell decreases of the cells in the G1-phase and an increases in S-phase, indicating MET promoted G1 phase cell entry into the S phase. Meanwhile, we found that overexpression of MET significantly rescued G0/G1-phase arrest induced by LINC01510 knockdown in LoVo and SW620 cells. The cell cycle regulatory proteins, CDK2 and P21, were upregulated in the MET groups, which was abrogated by LINC01510 knockdown (Fig. 6b). Taken together, these results demonstrated that LINC01510 function in cell cycle regulation at least in a MET-dependent manner.

## Discussion

Several studies have focused on the effect of long noncoding RNAs abnormally expressing in cancer and the association between noncoding RNAs and cancer development and progression in humans [25–27]. Our previous work has shown that long noncoding RNA GHET1 was significantly increased in CRC and promoted CRC cell proliferation and invasion [28]. In this study, for the first time, we found that LINC01510 was significantly increased in CRC tissues and cells compared with that in normal control. Knockdown of the expression of LINC01510 dramatically inhibited cell growth and the cell cycle transition from the G1-phase to the S-phase in CRC cells. The inhibition of proliferation and cell cycle arrest induced by LINC01510 was reversed by MET. These results indicated that LINC01510 was an important lncRNA that contributes to CRC growth and might act as a therapeutic target for the disease.

LncRNA has been identified as playing a critical role in various cancers and regulating various biological functions. For example, LncRNA SLC25A25-AS1 has been proved to be a tumor suppressor that inhibites CRC cell proliferation and chemoresistance [29]. LncRNA AFAP1-AS1 has been identified as a new lncRNA that regulates the cell cycle of CRC cells and plays a key role in cancer progress [30]. Induction of cell cycle arrest is an important mechanism by which tumor growth suppressed. Notably, we demonstrated that LINC01510 knockdown significantly inhibited cell growth with downregulation of the expression of anti-apoptosis protein Bcl-2 and the increase apoptosis protein bax and caspase3. We also revealed that silencing of LINC01510 inhibited the cells moving from the G1-phase into the S-phase. P21 was reported as a downstream target of P53 in the apoptosis pathway, inhibited cell proliferation by suppressing the kinases critical for cell cycle progression [20, 21, 31]. CDK2 bound to cyclinA to promote DNA duplication and suppressing the expression of CDK2 often induced cell cycle arrest [22, 23]. It has been reported that knockdown of P21 and CDK2 prevents the progress of CRC. We further demonstrated that LINC01510 knockdown significantly inhibited the protein expressions of P21 and CDk2. Thus, it was reasonable that LINC01510 could regulate cell cycle progress by suppressing P21 and CKD2 protein expression.

lncRNA–mRNA gene pairs could modulate tumorigenesis through their involvement in many biological processes. These lncRNAs perform the important function role in cancer by regulating their neighboring protein-coding genes, which resembles lncRNA-HIT and protein-coding gene ZEB1 [32]. lncRNA HIT000218960 was reported to promote thyroid cancer progress by upregulating the neighboring protein-coding gene HMGA2 [33]. MET has been found to be a tumor promoter in several types of cancer related to tumor growth, including CRC. It was reported that the protein-coding gene MET was upregulated in CRC and might be potentially used for in future prognostic or predictive models as a marker of CRC cancer [16, 17]. Higher levels of MET showed promoted cell proliferation, invasion and tumor budding in CRC [34]. In the present study, for the first time, we revealed that the expression of LINC01510 was positively associated with MET. We further demonstrated that LINC01510 knockdown dramatically inhibited the expression of MET. Moreover, LINC01510 knockdown induced the suppression of cell growth and cell cycle transition from the G1 to the S phase of CRC cells was reversed by MET. However, the exact mechanism of the lncRNA LINC01510 as an enhancer lnRNA to regulate MET-mediated progression of CRC still needs to be further investigated.

## Conclusion

Our data showed that LINC01510 was overexpressed in CRC tissues and cells related to clinicopathological grade and stage. Knockdown of LINC01510 inhibited cell growth and cell cycle progression through in a MET-dependent manner. LINC01510-MET might also be a potential therapeutic target for CRC.
